# Immunological and Viral Determinants of Dengue Severity in Hospitalized Adults in Ha Noi, Viet Nam

**DOI:** 10.1371/journal.pntd.0000967

**Published:** 2011-03-01

**Authors:** Annette Fox, Le Nguyen Minh Hoa, Cameron P. Simmons, Marcel Wolbers, Heiman F. L. Wertheim, Pham Thi Khuong, Tran Thi Hai Ninh, Trinh Thi Minh Lien, Nguyen Thi Lien, Nguyen Vu Trung, Nguyen Duc Hien, Jeremy Farrar, Peter Horby, Walter R. Taylor, Nguyen Van Kinh

**Affiliations:** 1 Oxford University Clinical Research Unit, Wellcome Trust Major Overseas Programme, Hanoi, Socialist Republic of Vietnam; 2 Centre for Tropical Medicine, Nuffield Department of Clinical Medicine, University of Oxford, Oxford, United Kingdom; 3 South East Asia Infectious Diseases Clinical Research Network, Jakarta, Indonesia; 4 National Hospital for Tropical Diseases, Hanoi, Socialist Republic of Vietnam; Southwest Foundation for Biomedical Research, United States of America

## Abstract

**Background:**

The relationships between the infecting dengue serotype, primary and secondary infection, viremia and dengue severity remain unclear. This cross-sectional study examined these interactions in adult patients hospitalized with dengue in Ha Noi.

**Methods and Findings:**

158 patients were enrolled between September 16 and November 11, 2008. Quantitative RT-PCR, serology and NS1 detection were used to confirm dengue infection, determine the serotype and plasma viral RNA concentration, and categorize infections as primary or secondary. 130 (82%) were laboratory confirmed. Serology was consistent with primary and secondary infection in 34% and 61%, respectively. The infecting serotype was DENV-1 in 42 (32%), DENV-2 in 39 (30%) and unknown in 49 (38%). Secondary infection was more common in DENV-2 infections (79%) compared to DENV-1 (36%, p<0.001). The proportion that developed dengue haemorrhagic fever (DHF) was 32% for secondary infection compared to 18% for primary infection (p = 0.14), and 26% for DENV-1 compared to 28% for DENV-2. The time until NS1 and plasma viral RNA were undetectable was shorter for DENV-2 compared to DENV-1 (p≤0.001) and plasma viral RNA concentration on day 5 was higher for DENV-1 (p = 0.03). Plasma viral RNA concentration was higher in secondary infection on day 5 of illness (p = 0.046). We didn't find an association between plasma viral RNA concentration and clinical severity.

**Conclusion:**

Dengue is emerging as a major public health problem in Ha Noi. DENV-1 and DENV-2 were the prevalent serotypes with similar numbers and clinical presentation. Secondary infection may be more common amongst DENV-2 than DENV-1 infections because DENV-2 infections resulted in lower plasma viral RNA concentrations and viral RNA concentrations were higher in secondary infection. The drivers of dengue emergence in northern Viet Nam need to be elucidated and public health measures instituted.

## Author Summary

Dengue is estimated to affect 50 million people each year and can occur as explosive outbreaks that overwhelm health systems. Despite significant advances the available knowledge is not sufficient to predict the outcome of individual infections or the occurrence of epidemics. Studies from low dengue transmission settings are lacking but offer the potential to better understand the contribution of age, primary versus secondary infection and serotype because there are likely to be more adult and primary infection patients and fewer serotypes circulating compared to high transmission settings. This is the first reported study of clinical dengue in Ha Noi, the largest urban area of Northern Viet Nam. Records kept by the Preventive Medicine Center indicate that <2500 clinical dengue cases attended government health care facilities in Ha Noi each year from 1999 until 2007. Patients in Ha Noi were older than in high transmission settings, the contribution of primary infection to overt and severe illness was greater and associations between serotype, plasma viral RNA concentration and overt and severe illness were distinct. The dengue situation in Ha Noi provides an opportunity to further examine the roles of serotype and prior immunity in dengue severity and epidemic emergence.

## Introduction

Dengue virus (DENV) infections range in severity from asymptomatic to a syndrome characterized by a haemorrhagic tendency and vascular permeability [1]. The events that precipitate endothelial cell dysfunction and vascular leak are incompletely understood. Numerous studies including several prospective cohorts [2], [3], [4] demonstrate that the risk of severe dengue is higher during a secondary infection with a new serotype in children [5], [6], [7]. Severe dengue has also been associated with high viral loads [8], [9], [10], [11], [12], prolonged viremia [13] and high NS1 antigen levels [12], [14]. At sub-neutralizing concentrations, dengue specific antibodies can enhance dengue virus infection of mononuclear phagocytes [15]. In addition, antibodies to pre-membrane protein appear to enhance infection of all serotypes, even when present at high concentration [16]. It has therefore been proposed that antibody-dependent enhancement (ADE) of viremia is a risk factor for severe dengue [1]. However, ADE may not fully account for severe dengue as several studies have found no association between severity and secondary infection in adults [7], [17], [18], [19], or severity and viral load [20], [21], [22], [23]. In one study dengue virus titers were higher prior to defervescence in patients with secondary infection [8] but most other studies have found that titers are either similar or higher in primary infection [10], [17], [20], [24]. Demonstration of a link between enhancing antibody levels, viral load and disease severity in humans also remains elusive.

The emerging picture is that multiple factors including prior immunity, viral load, age of the patient and infecting serotype and genotype may contribute to the severity of dengue infection [2], [3], [25], [26] but the nature of these interactions remains unclear. Dengue pathogenesis has largely been studied in dengue hyper-endemic regions where analysis is “confounded by a multiplicity of preexisting immunity patterns coupled with co-circulation of multiple serotypes” [3], [27]. Studies in low transmission settings, where few dengue serotypes circulate and primary infection in adults is common, potentially offer an opportunity to better identify factors associated with severity across serotype and immunity groups. We conducted a prospective study in Ha Noi, Viet Nam, to examine the association between primary and secondary infections, serotype, plasma viral RNA concentration, and the development of dengue haemorrhagic fever (DHF) in a low transmission setting.

## Methods

### Patient recruitment and clinical investigations

The protocol for this study was approved by the scientific and ethical committees at the National Hospital of Tropical Diseases and The Oxford University Tropical Research Ethics Committee (OXTREC). Patients provided written informed consent to participate in this study.

Patients were eligible for recruitment if they were admitted to the National Hospital of Tropical Diseases (NHTD) in Ha Noi, Viet Nam between September and November 2008 with a clinical diagnosis of dengue according to the WHO criteria [28]. These criteria were fever plus two or more of the following: headache; retro-orbital pain; myalgia/arthralgia; rash; bleeding or leukopenia. The study protocol included children but the number attending the National Hospital of Pediatrics (NHP) in Ha Noi during the study period was too low to warrant investigation. The NHTD is a 160 bed tertiary care center for adult patients with infectious diseases and also serves as a referral center for dengue in Northern Viet Nam. The NHP is the coordinating center for pediatric care in the country, receives patients from all Northern provinces and sees on average 40,000 in-patients and 350,000 out-patients per year.

Patients were examined daily during hospitalization by a dedicated team of physicians with experience in dengue diagnosis and treatment. Signs and symptoms of hemorrhage, capillary permeability and shock along with other relevant clinical data were prospectively recorded using standardized case record forms. Ultrasound of the chest and abdomen was performed at study enrolment and additionally when clinically indicated. Full blood counts were performed daily for at least 6 days during admission, as well as at discharge and approximately 10 days after discharge. Other investigations and clinical management were at the discretion of the attending physicians. CRFs and laboratory results were reviewed to identify patients that fulfilled the WHO criteria for dengue hemorrhagic fever (DHF), i.e. plasma leakage, plus thrombocytopenia and hemorrhagic signs [28]. Plasma leakage was said to be evident if pleural effusion and/or ascites were detected by ultrasound, or if a haematocrit during admission was ≥20% higher than at follow-up. If the patient did not attend follow-up, the average of follow-up values for males (n = 60, average  = 44) or females (n = 56, average  = 38) was used. This compares to a normal haematocrit value of 38% with a range of 35–41% set by the Ministry of Health of Viet Nam. Thrombocytopenia was defined as a platelet count less than 100,000/mm^3^.

### Dengue Diagnostics

An in-house IgM & IgG capture ELISA using antigens from DENV 1-4 and monoclonal antibodies provided by Venture Technologies (Sarawak, Malaysia) was performed as previously described [29]. A sample was considered IgM or IgG positive if the units were at least 6 times higher than the negative control sera. An internally controlled, serotype-specific, real-time reverse-transcriptase polymerase chain reaction (RT-PCR) assay [30] was used to identify the infecting serotype and determine viral RNA concentrations expressed as cDNA equivalents/ml of plasma. The sequences of the dengue serotype-specific primers and probes have been published previously [30]. They amplify and detect parts of the NS5 coding region that were first identified by Laue et al as being highly conserved within each dengue serotype [31]. The assay limit of detection was 10 cDNA equivalents per reaction. Dengue NS1 antigen was detected using a commercial ELISA (BIO-RAD Platelia™ Dengue NS1 Ag) according to the manufacturer's instructions. A diagnosis of confirmed dengue was made using a previously described reference algorithm [32] that has been adapted to include NS1 ELISA and remove the indirect recombinant membrane protein ELISA, which was not used in this study (Figure S1). Using this algorithm a patient is considered to have confirmed dengue if either RT-PCR or NS1 ELISA is positive, if there is an increase in the level of IgM detected by ELISA or an IgG ELISA conversion in the presence of a positive IgM ELISA. Serology was considered to be consistent with primary dengue infection if on or after day 6 of illness IgM levels were at least 1.78 times higher than IgG levels [33], or with secondary infection if IgM levels were less than 1.2 times higher than IgG levels.

### Data analysis

Illness day was calculated from the first date that the patient recalled having fever, which was assigned as day 1. Proportions were compared using odds ratios and Chi-Square or Fishers exact test when any expected cell count was less than 5. Continuous variables were presented as medians and interquartile ranges (IQR) and compared using Kruskal-Wallis and Mann Whitney tests. Data for study patients was compared to records kept by the Ha Noi Preventive Medicine Center (Ha Noi PMC), which includes age, gender, province and district of people attending government health care facilities with a clinical diagnosis of dengue by month and year.

We modelled dengue severity as depending on serological definition of prior infection/immunity and infecting serotype (and a potential interaction) using simple and multiple logistic regression analyses. The probability of a positive NS1 result and the log-10 plasma viral RNA measurements on day 5 of illness (i.e. the median illness day when patients were admitted) were modelled using logistic and linear regression models, respectively, depending on serological definition of prior infection, infecting serotype and dengue severity. In a sensitivity analysis, the model was additionally adjusted for age and gender. The time from illness onset to the first undetectable NS1 and viral RNA measurement, respectively, were modelled using Weibull accelerated failure time regression models for interval censored data, i.e. patients were treated as reaching undetectable levels in the interval between their last positive and their first undetectable measurement and patients for whom the first measurement was undetectable were treated as first reaching undetectable levels between illness day 1 and the day of this first measurement.

Analyses were performed with the statistical software R version 2.9.1 [34] and SPSS for Windows, Rel. 14.0.0.245, 2005 (SPSS Inc. Chicago IL.).

## Results

### Characteristics of patients admitted with confirmed dengue

158 of 206 eligible patients consented and enrolled in an 8 week period commencing on September 16 2008. During this time approximately 1240 clinical dengue cases attended government health care facilities in Ha Noi out of a total of 2371 for the whole of 2008 of which 975 (41%) were admitted to NHTD. 139 patients were from Ha Noi province, 11 were from 4 neighboring provinces and 6 were from more distant provinces, the farthest being Nhge An, Son La and Quang Ninh which are more than 100 km from Ha Noi. None of the patients were from provinces north of Ha Noi. Laboratory diagnosis of dengue was made for 130 patients. A further 18 with probable dengue were not included in this analysis as these were recruited significantly later in their illness and 61% had already defervesced. 26% of all enrolled patients and 23% of those with confirmed dengue had been transferred from another hospital. The age and gender distribution of confirmed dengue patients (Table 1) were similar to that for dengue cases attending any government health care facility in Ha Noi in 2008 (median age 23 years, IQR 18–31, 52% male). The geographic distribution was also similar to that for all reported cases in Ha Noi (data not shown). Amongst confirmed cases serology was indicative of secondary infection in 61% and of primary infection in 34% (Table 1). The infecting serotype could be defined by real-time-RT-PCR for 81 patients of which 52% had DENV-1 and 48% had DENV-2 (Table 1). Viral RNA could not be detected by RT-PCR in 49 confirmed dengue patients. The median admission day for these patients was 1 day later than for those with virus RNA detectable by RT-PCR (Table 1). The E genes of 9 DENV-2 (GenBank: GU908512- GU908520) and 20 DENV-1 (GenBank: HQ591537-HQ591556) viruses were sequenced and belong phylogenetically to the Asian 1 genotype and Genotype I, respectively (unpublished findings). Secondary infection was significantly more common in DENV-2 patients (79%) compared to DENV-1 patients (36%, p<0.001). Age, sex and course of illness were similar across serotype and serology subgroups (Table 1). Most patients (91, 70%) were classified as dengue fever (DF) and 36 (28%) developed DHF, of which 5 were classified as grade I, 30 as grade II and 1 as grade III. All patients were well at discharge except one patient who was transferred to the surgical hospital.

**Table 1:** Characteristics of confirmed cases categorized by prior immunity and serotype.

Patient Group		n (%)	% male	age	Admission day	Days febrile	Days in hospital	DHF n (%)
**All confirmed dengue**		130	53	23.5 (21–31)	5 (4–6)	5 (5–6)	5 (4–6)	36 (28)
**Primary serology** *		44 (34)	61	23 (21–32)	5 (4–6)	5 (4–6)	5 (4–6)	8 (18)
**Secondary serology**		79 (61)	48	24 (20–31)	5 (4–6)	5 (5–6)	5 (4–7)	25 (32)
**DENV-1**		42 (32)	57	24 (21–32)	4 (4–5)	5 (4–6)	6 (5–7)	11 (26)
**DENV-2**		39 (30)	54	23 (21–40)	4 (4–5)	5 (5–6)	5 (4–7)	11 (28)
**Serotype-unknown**		49 (38)	49	23 (20–30)	5 (5–6)	6 (5–6)	5 (4–6)	14 (29)
**DENV-1**	**Primary** *	24 (18)	62	23.5 (21–32)	4.5 (4–6)	5 (4–6)	6 (4–6)	5 (21)
	**Secondary**	15 (11)	53	24 (22–32)	4 (4–6)	5 (5–6)	6 (5–7)	5 (33)
**DENV-2**	**Primary**	8 (6)	62	23 (22–26)	4.5 (4–6)	5 (4–6)	5 (4–6)	2 (25)
	**Secondary**	31 (24)	52	25 (20–42)	4 (4–5)	6 (5–6)	5 (4–7)	9 (29)
**Serotype-unknown**	**Primary** *	12 (9)	58	22.5 (20–33)	5 (4–6)	6 (5–7)	4 (3–5)	1 (8)
	**Secondary**	33 (25)	42	24 (20–28)	4 (4–5)	5 (5–6)	5 (4–6)	11 (33)

*Serology to define prior infection status was indeterminate for 7 patients (5%) including 3 DENV-1 and 4 serotype-unknown patients.

### Characteristics of patients with DHF

**Figure 1:**
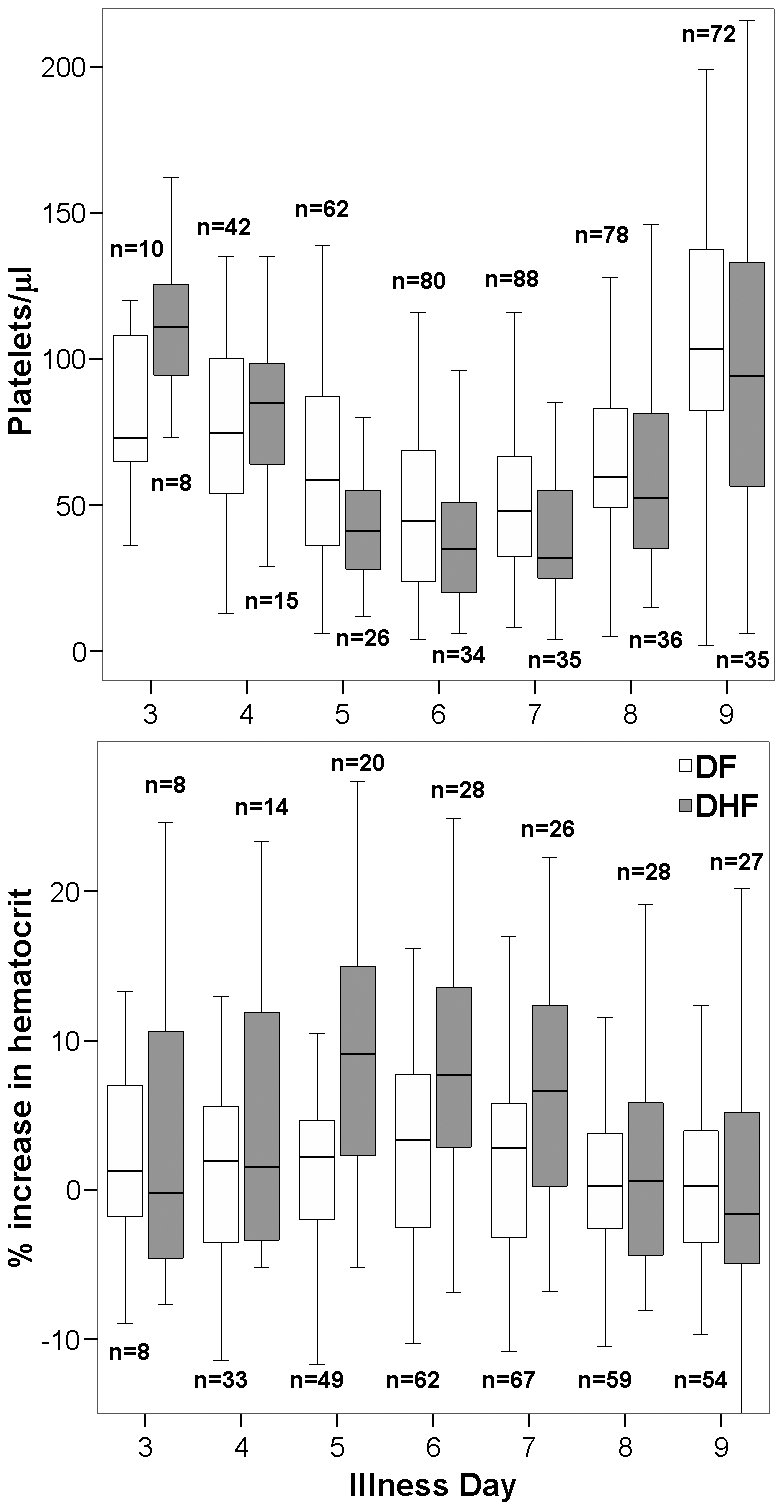
Daily platelet and haematocrit measurements in DF and DHF patients. The number of DF (94 in total) and DHF patients (36 in total) assessed each day is shown above or below each box. Haematocrits were assessed on at least 4 days for 125 of 130 confirmed dengue patients.

In patients with DHF platelet counts fell to their lowest levels and haematocrits increased by the greatest percentage over baseline on days 5–7 (Figure 1). DHF rates were similar for DENV-1 and DENV-2 patients (Table 1). There was a non-significant trend of higher DHF rates in patients with secondary compared to primary infection (Odds Ratio 1.96, 95% CI 0.80 – 4.85, p = 0.14). Results were consistent when including both serotype and serological definition of primary versus secondary infection in a logistic model and after adjusting for age and sex (data not shown), and there was no evidence of an interaction between serotype and primary/secondary infection status in the development of DHF (Likelihood ratio p = 0.48).

### Association between serotype, serology and NS1

**Figure 2:**
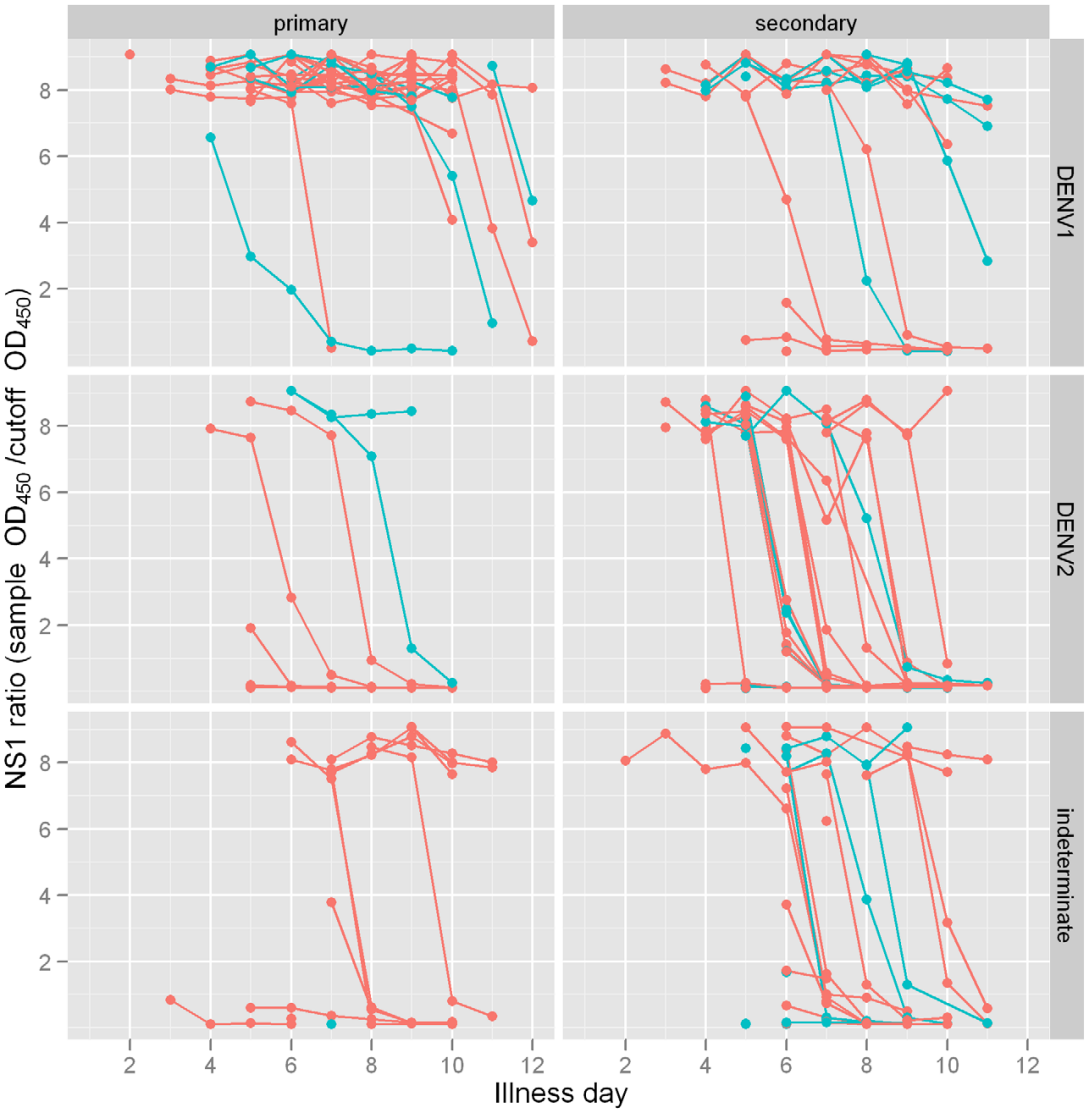
NS1 measurements in DF and DHF patients stratified by serotype and primary versus secondary infection. Plasma samples collected daily during admission and at follow-up from DF (pink) and DHF (blue) patients were tested by NS1 ELISA and results presented as OD_450_ sample/OD_450_ cutoff control.

The proportion NS1 positive on day 5 of illness was significantly higher for DENV-1 compared to DENV-2 patients and the time to undetectable NS1 was shorter for DENV-2 (Table 2, Figure 2). The proportion NS1 positive on day 5 was similar for patients with primary and secondary infection but the time to undetectable was shorter for secondary infection. There was no evidence of an interaction between serotype and primary/secondary infection status (Likelihood ratio  = 0.24) and effects were consistent when the analysis was additionally adjusted for age and gender. There was also no evidence of any association between NS1 and DHF (Table 2).

**Table 2:** Multiple regression models for prediction of NS1 detection.

Covariate	Undetectable NS1 on day 5		Time to undetectable NS1	
	OR (95%CI) *	p-value	Effect (95%CI)*	p-value
DENV-2 versus DENV-1	13.88 (1.40 to 137.12)	0.02	0.51 (0.41 to 0.63)	<0.001
Unknown versus DENV-1	31.13 (2.54 to 381.97)	0.01	0.61 (0.50 to 0.75)	<0.001
Secondary versus primary	0.74 (0.14 to 3.86)	0.72	0.82 (0.69 to 0.98)	0.03
DHF versus DF	1.57 (0.36 to 6.86)	0.55	0.97 (0.82 to 1.15)	0.74

Logistic regression for modeling the probability of undetectable NS1 on day 5, Weibull regression for time to undetectable NS1. Plasma samples from all (130) confirmed dengue patients were assessed but only 67 had plasma collected on day 5 of illness including 30 DENV-2, 27 DENV-1 patients and 24 with primary infection and 40 with secondary infection.

*Multiplicative effect, i.e., the odds for DENV-2 patients to have undetectable NS1 on day 5 is estimated to be by a factor of 13.88 higher than for DENV-1 and the time to undetectable NS1 for patients with DENV-2 is estimated to be shorter by factor of 0.51.

### Association between serotype, serology and plasma viral RNA concentration

**Figure 3:**
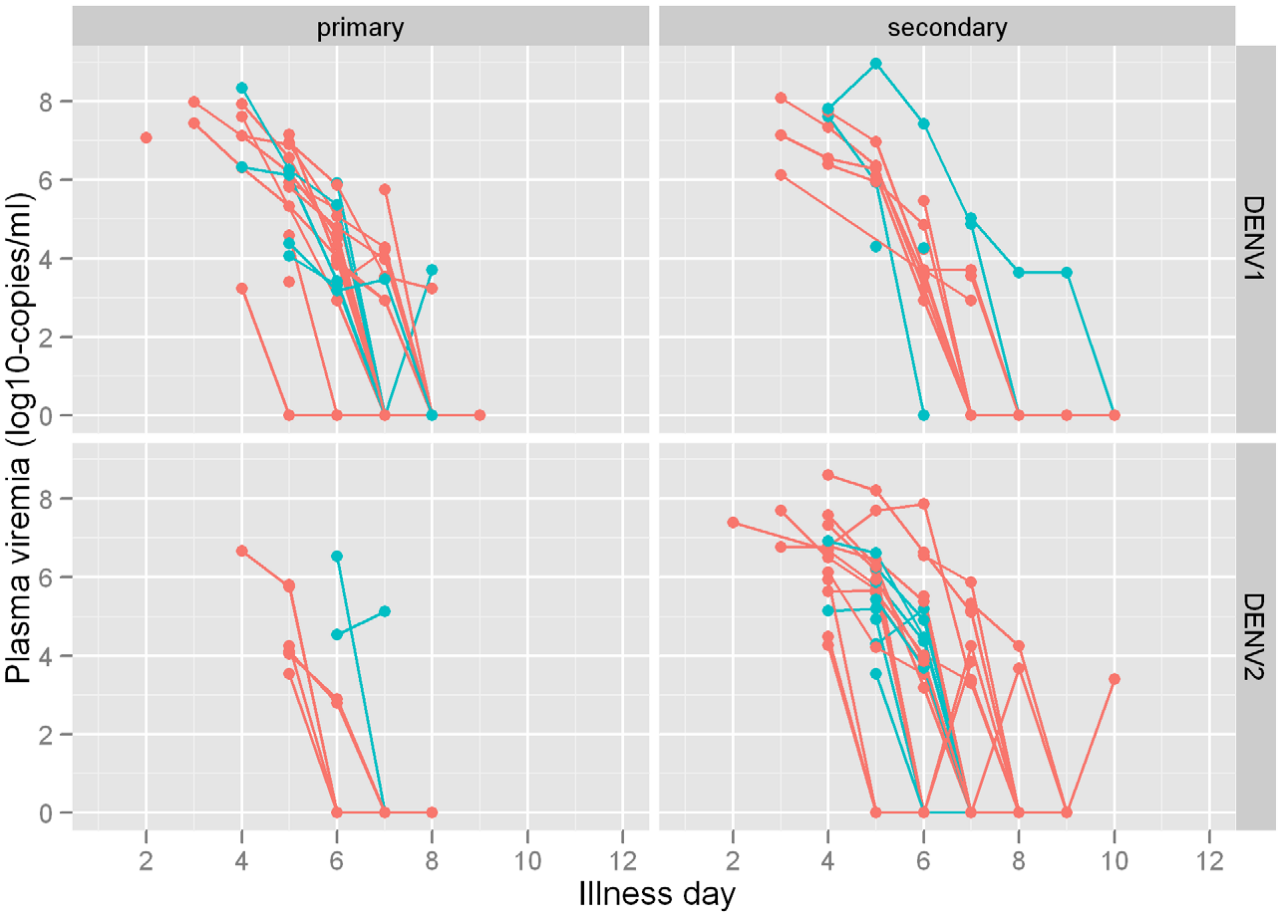
Viral RNA concentration in DF and DHF patients stratified by serotype and primary versus secondary infection. Plasma samples collected daily during admission from DF (pink) and DHF (blue) patients were tested by RT-PCR for viral RNA and results expressed as Log10 cDNA equivalents/ml of plasma.

Log10 viral RNA concentration in plasma on day 5 was estimated to be 0.96 lower for DENV-2 than for DENV-1 patients and the time taken until viral RNA was undetectable in plasma was estimated to be 0.89 times shorter for DENV-2 (Table 3, Figure 3). Plasma viral RNA concentration on day 5 was significantly higher in patients with secondary infection, but the time taken until viral RNA was undetectable in plasma was not significantly different. Results were consistent after adjusting for age and sex (data not shown) and likelihood ratio tests showed no evidence of interaction between serotype and primary/secondary infection status for either viral RNA concentration on day 5 (p = 0.96) or time taken until viral RNA was undetectable (p = 0.85). We couldn't establish a clear association between DHF and plasma viral RNA concentration on day 5 or time taken until viral RNA was undetectable in plasma.

**Table 3:** Multiple regression models for prediction of plasma log10 viral RNA concentration and time to undetectable.

Covariate	log10-viral RNA concentration on day 5		Time until viral RNA is undetectable in plasma	
	Effect (95%CI) *	p-value	Effect (95%CI)**	p-value
DENV-2 versus DENV-1	−0.96 (−1.78 to −0.13)	0.02	0.89 (0.83 to 0.96)	0.001
Secondary versus primary infection	+0.86 (0.01 to 1.71)	0.046	1.06 (0.99 to 1.14)	0.12
DHF versus DF	−0.21 (−1.03 to 0.61)	0.61	1.03 (0.96 to 1.11)	0.38

Linear regression for modeling the viral RNA concentration in plasma on day 5, Weibull regression for time to undetectable viral RNA. Plasma samples from all (130) confirmed dengue patients were assessed but only 67 had plasma collected on day 5 of illness as described in Table 2.

*Additive effect.

**Multiplicative effect.

## Discussion

Hospitalized dengue patients in Ha Noi in 2008 were predominantly adults with high rates of primary infection compared to Southern Vietnam [35] and other hyper-endemic regions [36], [37]. DENV-1 and DENV-2 were the only serotypes identified, consistent with national dengue surveillance data for Ha Noi (Le Quynh Mai, personal communication), whereas in Southern Viet Nam all serotypes were detected during clinical dengue surveillance in 2008 but DENV-1 was predominant [38]. Overall case numbers and clinical presentation were similar for DENV-1 and DENV-2. However, while primary infection predominated amongst DENV-1 patients suggesting that a substantial proportion of adults are dengue naïve, secondary infection predominated in DENV-2. This suggests that primary DENV-2 infections may be less likely to present clinically.

A limitation of this study is that patients were recruited from only one hospital in Hanoi. However, the patients studied represented ∼12% of dengue cases seeking treatment for dengue at government health care centers in Ha Noi during the study period and had similar epidemiology. We did not include children as originally intended because only 5 were admitted to the National Hospital of Pediatrics with clinically suspected dengue while the study was being conducted. This may be expected if transmission is low such that secondary infections mainly occur in adulthood. However it is not clear why children with primary infection do not present given that 34% of adult patients had primary infection. Others report that DHF rates during primary infection are higher for adults and suggest that primary infection is more severe in adults [39]. Patients that admitted late were not precluded from this study in order to obtain a comprehensive description of clinical dengue, however this limited our ability to detect viral RNA in plasma and determine peak concentrations. The infecting serotype was unknown for 38% of confirmed dengue patients most of whom admitted 5 to 6 days after illness onset, and by day 6 viral RNA could be detected in only 39 of 100 confirmed patients tested. We suspect that DENV-2 will be the predominant infecting serotype in this group because viral RNA clearance from plasma was faster for DENV-2 than for DENV-1 patients in our study.

Similar to the findings of this study, records kept since 1988 indicate that Dengue case numbers have been low in Ha Noi and that dengue has predominated in adults (Horby P. et al, in preparation). This may not reflect transmission because the proportion of infections that present clinically can be low and the proportion and age-distribution depends on the prevalent serotypes and the age-associated prevalence of past infection with each serotype [3], [40], [41]. However, the age of clinical dengue cases generally increases with decreasing transmission intensity [42], and the epidemiology of hospitalized dengue in this study is similar to that in Singapore where transmission has decreased due to effective vector control but the age and proportion of cases with primary dengue has increased, presumably because adults are more prone to present clinically upon primary infection [40].

Others have reported that primary DENV-2 infections are rarely symptomatic [6], [36], [37], [43], [44]. The reason for this has not been established but our data shows that viral RNA concentration is low and NS1 detection brief in the plasma of DENV-2 compared to DENV-1 patients, factors that could be considered important in disease pathogenesis leading to severe dengue. Furthermore, the relatively high prevalence of secondary DENV-2 coincided with higher plasma viral RNA concentrations in secondary infection. It is important to note that the DENV-2 viruses sequenced in this study belonged to the Asian I genotype, which has been associated with more severe disease compared to the American genotype [26] and with higher plasma viral RNA concentrations compared to Asian/American genotype [38]. While the results suggest that primary infection with DENV-1 is more likely to lead to clinically overt disease than with DENV-2, we can not exclude the possibility that secondary infection contributes to overt DENV-1 or the possibility that DENV-2 infections are more likely to be enhanced than DENV-1 infections. The latter has been suggested elsewhere [3] and is supported by several studies showing that in children with secondary infection DHF is more common for DENV-2 compared to DENV-1 [4], [36]. DHF was approximately twice as common in secondary compared to primary infection in our cohort, but the number of patients with DHF was small and this did not reach statistical significance or permit analysis within each serotype. However, in a study of hospitalized patients in Thailand the association between secondary infection and DHF was greater for DENV-2 than DENV-1 because DHF was less common in DENV-2 compared to DENV-1 during primary infection [34]. As in our study, this suggests that primary DENV-2 infections may be less virulent than DENV-1. As discussed above, we suspect that DENV-2 would have been the predominant infecting serotype amongst confirmed dengue patients in which the infecting serotype was unknown. NS1 measurements and prior infection status were similar for serotype-unknown and DENV-2 patients and distinct from DENV-1 patients providing further indication that DENV-1 may be distinct in terms of virulence during primary infection.

There was a non-significant trend of increased DHF in secondary infection, but 22% of DHF cases had primary infection. Thus secondary infection was not essential for DHF in this cohort. Secondary infection was associated with higher viral RNA concentration in plasma on day 5 of illness, but we did not find an association between viral RNA concentration and DHF. Interpretation of the effect of viral RNA concentration on DHF in our patients is limited by the relatively low proportion that developed DHF and perhaps also the high proportion that presented after day 3 of illness. Several of the studies that find an association between viremia and DHF recruit children within the first 3 days of illness and suggest that DHF is positively associated with peak viremia [8], [9], [11]. It remains controversial whether virus clearance times also differ. In one study clearance of infectious virus determined by mosquito inoculation was faster in children with DHF compared to DF [8] but studies of adults with DENV-2 or DENV-3 infection [10], [13] and children with DENV-2 infection [24] have found longer times to virus RNA or virus-RNA containing immune complex clearance amongst those with DHF[10], [13]. It is also possible that we did not detect an association between DHF and plasma viral RNA concentration because a relatively high proportion of our patients had primary DENV-1 and studies where DHF has been associated with viremia rarely include primary DENV-1 [9], [10], [11], [13], [14], [24] or reported that there was no association in patients with primary DENV-1 [8]. The contribution of plasma viral RNA concentration to the development of DHF may be not be discernable in primary DENV-1 because plasma viral RNA concentration is generally high in DENV-1, but this would imply that high viral RNA concentration alone is not sufficient to cause DHF.

The time until NS1 was undetectable was longer for DENV-1 compared to DENV-2, similar to findings of an earlier study in Southern Viet Nam [32]. We previously suggested that this reflected a predominance of primary infection in DENV-1 and that NS1 clearance is faster in secondary infection due to sequestration by IgG [32], [45]. In the current study there were sufficient primary cases for a stratified analysis, which demonstrated that serotype is the main determinant of the sensitivity of NS1 tests, and this should be considered when interpreting NS1-based diagnostics.

In conclusion our results indicate an association between secondary infection and clinically overt DENV-2 infection. Higher plasma viral RNA concentration in secondary infection may underlie the association between secondary infection and overt DENV-2. We could not detect an association between DHF and secondary infection or plasma viral RNA concentration but this may be due to the relatively high proportion of patients with primary DENV-1, a situation that may change if dengue emerges and the proportion and age of the population that is dengue naïve declines.

The number of countries affected by dengue has increased six-fold in the last 30 years with potential for further spread through temperate, subtropical and tropical areas [46]. The Ha Noi Preventive Medicine Centre reported a 7-fold increase in the number of clinical dengue cases from 2008 to 2009 and this unforeseen epidemic overwhelmed the health system. A similar problem is faced in regions where dengue has been endemic for decades due to large multi-annual peaks in severe disease incidence [47]. Current understanding of the drivers of dengue epidemics is inadequate to predict their occurrence and inform public health prevention and preparedness measures. The dengue situation in Ha Noi provides an opportunity to further examine the roles of serotype infection sequence and prior immunity in dengue severity and emergence.

## Supporting Information

Figure S1 Dengue lab algorithm
(TIFF)
