# Supplementary figures and images for: Immunological and Viral Determinants of Dengue Severity in Hospitalized Adults in Ha Noi, Viet Nam
Source: PLoS Negl Trop Dis. 2011 Mar 1;5(3):e967. doi: 10.1371/journal.pntd.0000967 (PMC3046970; doi:10.1371/journal.pntd.0000967)

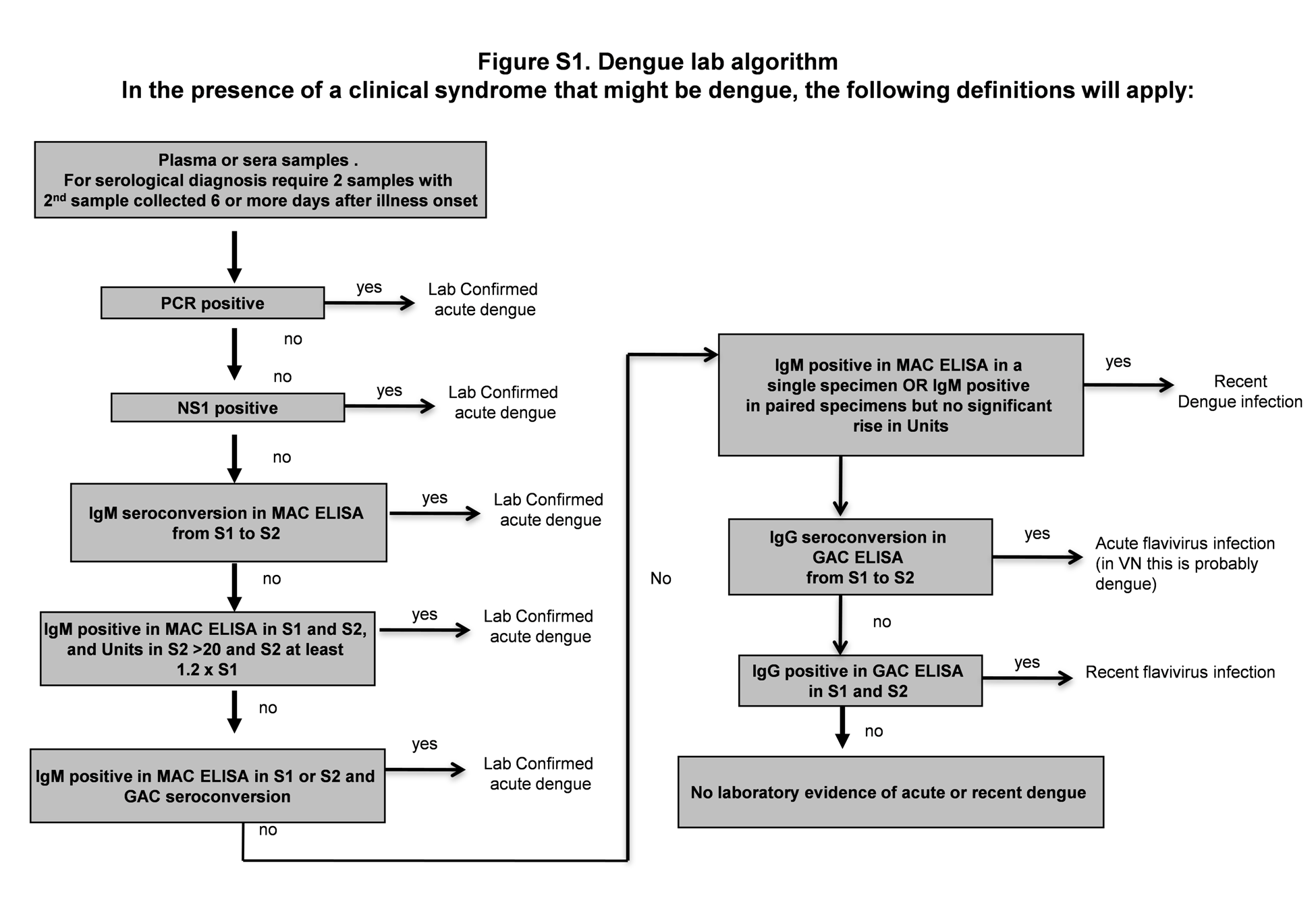

Supplement: Figure S1 — Dengue lab algorithm (TIFF) [file pntd.0000967.s001.tif]
